# Noonan syndrome and parasternal pericardiocentesis

**DOI:** 10.1007/s12471-025-01931-w

**Published:** 2025-02-20

**Authors:** Pitt O. Lim, May H. Ohn

**Affiliations:** Department of Cardiology, St George’s University Hospitals NHS Foundation Trust, London, UK

A 47-year-old woman with Noonan syndrome and pneumococcal pneumonia presented with cough, breathlessness, and hypotension caused by cardiac tamponade (Fig. 1a). Due to associated abdominal organomegaly, she underwent the recently described two-step parasternal pericardiocentesis approach [1]. The pericardial pressure was 17 mm Hg (normal < 4 mm Hg). About 1 L of serosanguinous fluid was withdrawn, resulting in immediate symptomatic relief and a doubling of the electrocardiographic R‑wave amplitude—an intuitive phenomenon not widely reported in the medical literature. The drain was removed the following day and a chest X‑ray showed pneumopericardium (Fig. 1b). Her CT scan confirmed the diagnosis and additionally revealed right basal lung consolidation and hepatosplenomegaly, which made subcostal pericardiocentesis challenging. Although there are no published cases of Noonan syndrome and percutaneous pericardiocentesis. The mechanism of pneumopericardium remains unclear but may result from pneumonic intrathoracic suction or potentially air leakage caused by the drain slipping out, exposing a side hole which is 7.5 cm from the tip [2]. Her follow-up echocardiogram however showed no tamponade physiology.

**Fig. 1:**
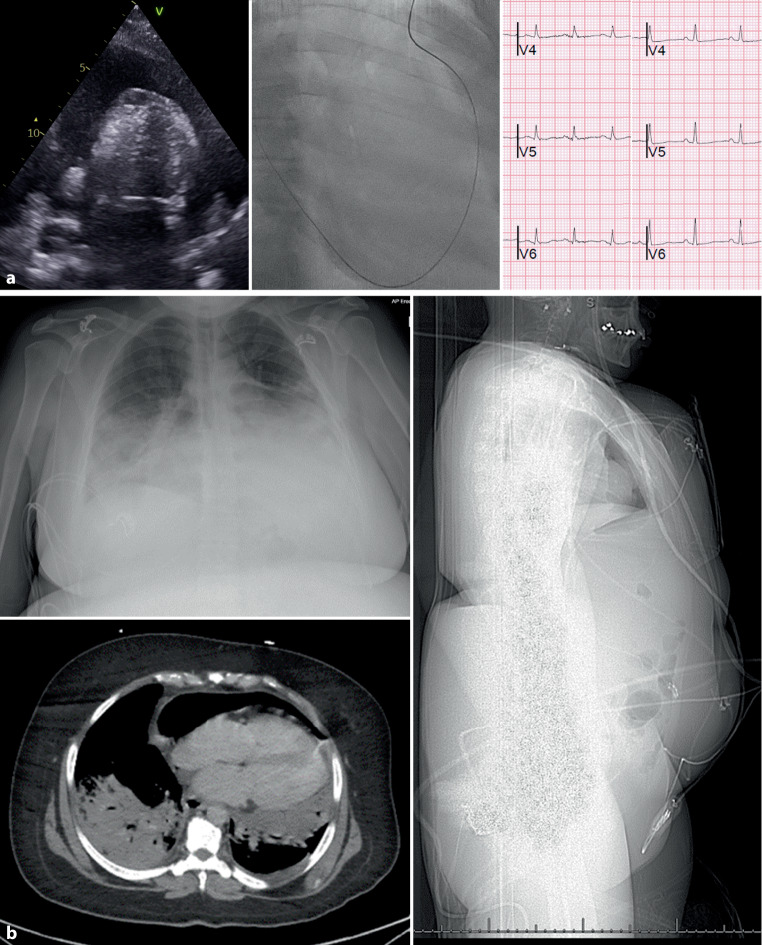
**a** *Left* Echocardiogram showing the large pericardial effusion causing cardiac tamponade. *Middle* Guidewire in the pericardial space using the 2‑step parasternal pericardiocentesis technique. *Right* Doubling of the electrocardiographic R wave amplitude following drainage of the pericardial effusion. **b** *Top left* Chest X‑Ray revealing pneumopericardium. *Bottom left* Coronal CT Scan demonstrating pneumopericardium, residual effusion, in addition to left basal lung consolidation. *Right *Sagittal CT Scan confirming hepatosplenomegaly, making subcostal pericardiocentesis inadvisable

## References

[1] Lim PO. Non-traumatic parasternal pericardiocentesis. BMJ Case Rep. 2023;16:e253728. 10.1136/bcr-2022-253728. 37080634 10.1136/bcr-2022-253728 PMC10124190

[2] Narins CR, Lee J, Cole M, et al. Pneumopericardium Following Pericardiocentesis. Am J Med. 2016;129:e181–2. 10.1016/j.amjmed.2016.03.033 27107923

